# Comprehensive Facial Skin Rejuvenation With Long‐Term Regular Intense Pulsed Light Therapy: A Real‐World Study

**DOI:** 10.1111/jocd.70691

**Published:** 2026-01-25

**Authors:** Birao Fan, Ruixing Yu

**Affiliations:** ^1^ Department Of Dermatology China‐Japan Friendship Hospital Beijing China

**Keywords:** acne, GAIS, intense pulsed light, real‐world study, rosacea, skin rejuvenation, VISIA imaging

## Abstract

**Background:**

Intense pulsed light (IPL) therapy is widely used for facial rejuvenation, targeting vascular, pigmentary, and textural changes. However, comprehensive, real‐world evidence evaluating long‐term, regular IPL treatment across multiple dimensions of skin improvement remains limited.

**Objectives:**

This study aimed to assess the efficacy and safety of long‐term, regular IPL therapy in improving facial erythema, pigmentation, and wrinkles, and to identify predictors of favorable response.

**Methods:**

This retrospective real‐world study included 236 patients who underwent six or more IPL sessions between 2020 and 2025. Patients were categorized as acne, rosacea, or cosmetic subjects. VISIA imaging quantified erythema, pigmentation, and wrinkle indices, while the Global Aesthetic Improvement Scale (GAIS) was used to assess aesthetic outcomes. Logistic regression identified predictors of good response.

**Results:**

Significant improvements were observed in erythema, pigmentation, and wrinkle indices after treatment (all *p* < 0.05). Regular treatment intervals and total number of sessions were independently associated with better outcomes (OR = 13.62 and 3.80, respectively, both *p* < 0.05). Patients with Fitzpatrick Type IV skin showed lower response rates (OR = 0.12, *p* = 0.001). VISIA analyses demonstrated quantifiable reductions in erythema and pigmentation areas, while wrinkles showed notable textural improvement. No severe adverse events occurred.

**Conclusions:**

Long‐term, regular IPL therapy effectively improves facial erythema, pigmentation, and wrinkles, confirming its value as a comprehensive rejuvenation strategy. Regular treatment intervals optimize cumulative effects, while objective imaging enhances precision in outcome evaluation. These findings support IPL as a safe, evidence‐based, and multidimensional approach to long‐term facial rejuvenation.

## 1 Introduction

In recent years, advancements in skin rejuvenation technologies have introduced various innovative methods to enhance treatment efficacy and precision. For instance, emerging techniques such as Laser‐Assisted Exosome Delivery with Fractional CO_2_ Laser and Sequential Fractional CO_2_ and 1540/1570 nm Lasers focus on novel approaches to treat erythema and pigmentation [1, 2]. These new methods provide complementary solutions that work alongside established therapies like IPL, which continues to be popular due to its broad‐spectrum light emission, enabling it to simultaneously target vascular and pigmentary abnormalities. Recent studies have focused on optimizing treatment parameters, such as fluence and pulse duration, for different skin types, which play a critical role in maximizing efficacy while minimizing side effects. Among the available modalities, intense pulsed light (IPL) therapy is particularly versatile, as its broad‐spectrum emission (500–1200 nm) enables simultaneous targeting of chromophores such as oxyhemoglobin and melanin, while inducing dermal remodeling [3, 4, 5]. Through selective photothermolysis, IPL can address vascular dilation, pigment irregularities, and superficial textural concerns in a single treatment paradigm [6].

Most published studies, however, have focused narrowly on single outcome domains—most often erythema or pigmentation—and have typically involved short‐term or single‐course interventions [7]. Few have systematically evaluated the integrated benefits of IPL on all three major dimensions of skin improvement under real‐world conditions. Moreover, long‐term consistency and treatment regularity are rarely quantified, despite their potential influence on cumulative outcomes. From a clinical standpoint, this evidence gap limits the development of standardized maintenance protocols for sustained rejuvenation.

In addition, objective imaging tools such as the VISIA Complexion Analysis System have made it possible to quantitatively assess treatment outcomes. By providing reproducible metrics for erythema, pigmentation, and wrinkles, VISIA imaging minimizes observer bias and enhances reliability in retrospective analyses [8, 9]. Integrating such imaging with validated clinical scoring systems such as the Global Aesthetic Improvement Scale (GAIS) enables a multidimensional evaluation of efficacy [10].

The present real‐world study therefore aimed to comprehensively evaluate long‐term, regular IPL therapy for facial rejuvenation across erythema, pigmentation, and wrinkle dimensions. Specifically, we sought to (1) quantify improvements using VISIA‐based imaging, (2) identify predictors of favorable clinical response, and (3) elucidate the impact of treatment regularity on cumulative outcomes.

## 2 Methods

### 2.1 Participants Enrollment

This study retrospectively collected a total of 236 patients who underwent IPL treatment six or more times in our hospital from January 2020 to September 2025. This includes three groups: acne, rosacea, and cosmetic subjects. Informed consent was obtained from all patients before study procedures were initiated. The inclusion criteria include: the patient's age is at least 18 years old, VISIA image records are kept for each treatment, and the adverse reaction investigation form after treatment is filled out truthfully. Exclusion criteria included a history of any other non‐pharmaceutical treatments for facial issues within 6 months before inclusion, an inability to refrain from tanning or using a tanning bed within the treatment process, any inflammatory skin condition or autoimmune skin disease in the target site, cancer and/or on cancer drug therapy, a history of keloid scarring, being pregnant, uncontrolled diabetes, or epilepsy.

### 2.2 Treatment Procedure

All patients underwent IPL therapy using the Lumenis M22 system (Lumenis Ltd., Yokneam, Israel). The treatment parameters were as follows:

*Fluence*: Fluence was tailored based on the patient's skin type and clinical condition, ranging from 12 to 18 J/cm^2^ for Fitzpatrick skin Types I–III, and 10 to 15 J/cm^2^ for Type IV.

*Pulse Duration*: Pulse duration was set between 5 and 20 ms depending on the clinical severity of erythema or pigmentation.

*Filters*: A 515–560 nm wavelength filter was used for most cases to target both vascular and pigmentary lesions. The filter was adjusted based on the clinical endpoint, with particular care for darker skin types to minimize adverse effects.

The desired endpoint was defined as mild erythema and transient darkening of pigmented lesions. Before each IPL session, a thorough consultation was conducted to assess the patient's skin condition and determine the appropriate treatment parameters. A thin layer of cool ultrasonic gel was applied to the treatment area to ensure optimal light coupling and provide epidermal protection. Both the practitioner and the patient wore appropriate wavelength‐specific protective eyewear.

Treatments were administered at regularly scheduled intervals. The first three sessions were conducted at 4–6 week intervals, with subsequent sessions spaced 6–8 weeks apart as the clinical condition improved. This adjustment allowed for adequate tissue recovery and aimed to consolidate therapeutic gains. Post‐treatment care included the application of a cooling pack for 15 min, and patients were instructed to use moisturizing cream and avoid sun exposure. As the clinical condition improved and the intensity of facial erythema diminished with successive treatments, the interval between subsequent sessions was progressively extended to 6–8 weeks. This protocol adjustment allowed for adequate tissue recovery and was designed to consolidate therapeutic gains while minimizing the risk of adverse effects [11, 12]. Postoperative care included the application of a cooling pack for 15 min, and all patients were instructed to use moisturizing cream topically and avoid sun exposure.

### 2.3 Image Acquisition and Quantitative Analysis

#### 2.3.1 Image Acquisition

Facial images of all patients were captured under standardized conditions using the VISIA Complexion Analysis System (Canfield Scientific Inc., USA). For each patient, two sets of images were acquired at baseline (pre‐treatment) and during each follow‐up visit (post‐treatment):

Standard photographs with polarized lighting were used to assess erythema. Specifically, the “Red Areas” images generated by the VISIA system, which employ cross‐polarized lighting to isolate and enhance the visibility of subcutaneous erythema and vasculature, were utilized for this purpose. Ultraviolet (UV) photographs were used to assess hyperpigmentation. These images, which reveal superficial and subclinical pigmented lesions by enhancing the contrast of melanin, were employed for the analysis of pigmented lesions. All images were acquired with consistent patient positioning, lighting, and camera settings to ensure comparability over time.

#### 2.3.2 Quantitative Image Analysis

Quantitative analysis of the erythema and hyperpigmentation was performed using ImageJ software (National Institutes of Health, USA, version 1.53 k). Measurements were executed with the Limit to Threshold option enabled. For each image, the Total Area (mm^2^) and Mean Gray Value of the erythema or hyperpigmentation were recorded.

In addition to the custom image analysis, the intrinsic “Wrinkle” analysis module of the VISIA system was utilized to quantitatively assess the improvement in skin texture. This automated tool employs a proprietary algorithm to generate a standardized “Wrinkle Index” score based on skin texture and shadow depth from the standard photographs. The Wrinkle Index scores at baseline and each follow‐up visit were recorded and compared.

### 2.4 Statistical Analysis

Statistical analysis was performed using SPSS Statistics, version 30.0. The Shapiro–Wilk test was used to assess the normality of data distribution. Since the quantitative data (Area and Mean Gray Value) are likely non‐normally distributed, pre‐ and post‐treatment values were compared using the non‐parametric Wilcoxon signed‐rank test. Data are presented as median and interquartile range (IQR). A two‐tailed *p* < 0.05 was considered statistically significant.

Treatment response was evaluated using the Global Aesthetic Improvement Scale (GAIS). Patients with GAIS ≥ 4 were defined as good responders, whereas those with GAIS < 4 were classified as poor responders. A binary logistic regression model was applied to identify factors associated with good response. Independent variables included age, sex, Fitzpatrick skin type, diagnosis, regularity of treatment, and number of IPL sessions. Odds ratios (OR) and 95% confidence intervals (CI) were calculated. Model performance was assessed using the area under the ROC curve (AUC) and McFadden's pseudo‐*R* ^2^. All analyses were performed using Python (statsmodels package). Statistical significance was set at *p* < 0.05.

## 3 Results

### 3.1 Patient Characteristics

A total of 236 patients were included, consisting of 41 males (17.4%) and 195 females (82.6%), with a mean age of 30 years (range: 22–38). Fitzpatrick skin Types III and IV comprised 77.5% and 22.5% of participants, respectively. Diagnoses included acne (15.3%), rosacea (23.3%), and cosmetic rejuvenation (61.4%). Regular treatment intervals were maintained by 78.0% of patients. Table 1 summarizes demographic and baseline data.

**TABLE 1:** Patients' demographics and baseline characteristics.

Gender, *n* (%)	
Male	41 (17.4)
Female	195 (82.6)
Age (years)	
Mean (CI)	30 (29.3, 30.7)
Min–max	22–38
Fitzpatrick skin type, *n* (%)	
Type III	183 (77.5)
Type IV	53 (22.5)
Concomitant dermatoses, *n* (%)	
Acne	36 (15.3)
Rosacea	55 (23.3)
Cosmetic subjects	145 (61.4)
Number of treatments, *n* (%)	
6	188 (79.7)
> 6	48 (20.3)
Frequency of treatments, *n* (%)	
Regular treatment	184 (78.0)
Irregular treatment	52 (22.0)

### 3.2 Quantitative Improvements in Erythema, Pigmentation, and Wrinkles

**FIGURE 1:**
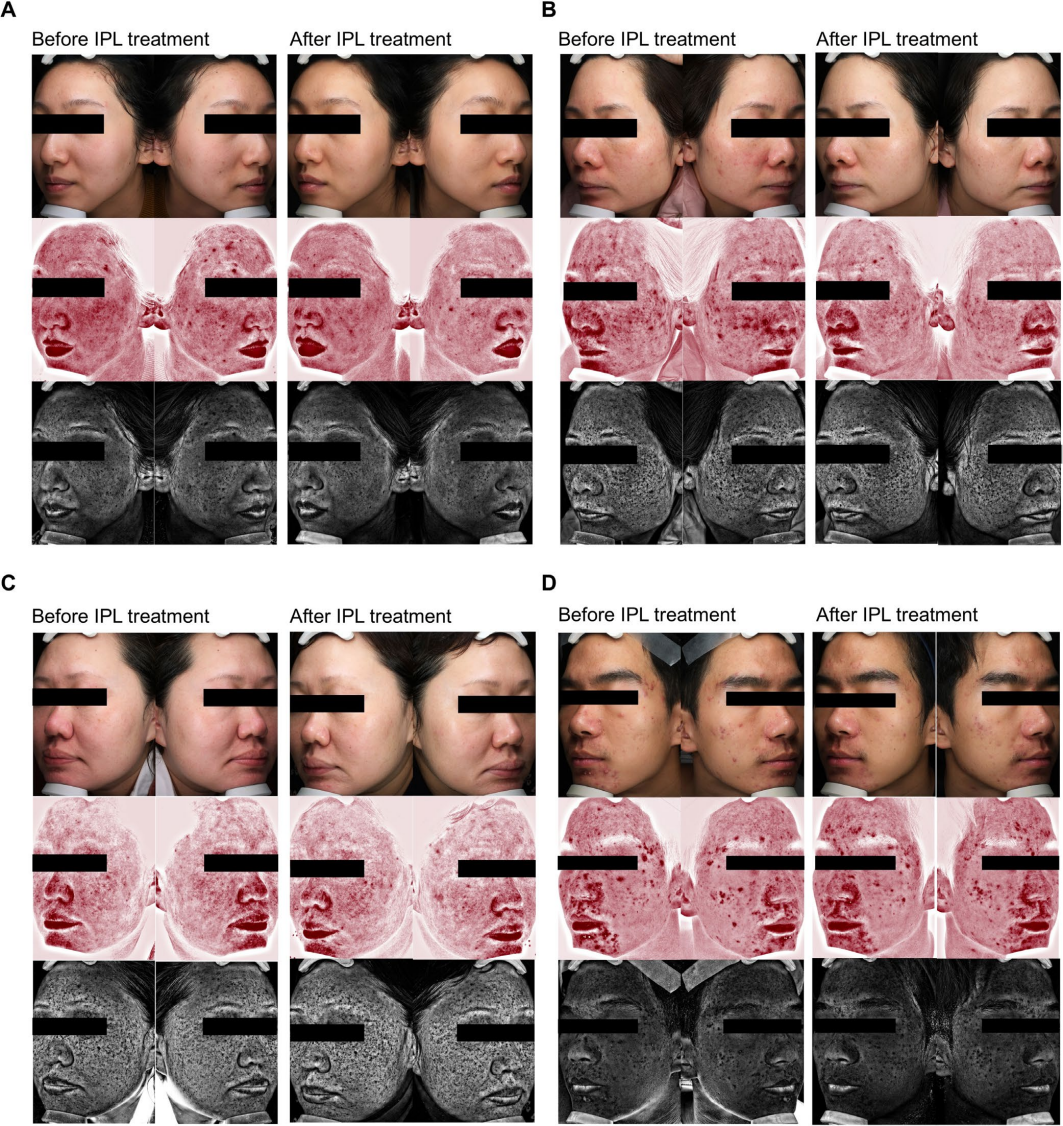
Comparison of split‐face VISIA images before and after IPL treatment of four subjects: (A) and (C) cosmetic subjects, (B) a rosacea patient, and (D) an acne patient.

Significant post‐treatment improvements were observed across all VISIA parameters (Table 2). In the regular treatment group, median erythema area decreased from 640.00 mm^2^ (IQR 405.79–916.83) to 372.40 mm^2^ (IQR 201.37–635.25, *p* < 0.001), while pigmentation area decreased from 577.54 mm^2^ to 370.61 mm^2^ (*p* < 0.001). Wrinkle index improved by a median of 123.5 (IQR 81.5–167.0, *p* < 0.001). These quantitative changes demonstrate IPL's multi‐target capacity to address vascular, pigmentary, and textural abnormalities concurrently. Representative VISIA images are presented in Figure 1, illustrating the stepwise improvements in erythema, pigmentation, and wrinkle reduction after six sessions.

**TABLE 2:** Quantitative data analysis of treatment outcomes.

	Group	Baseline	After 6th treatment	Improvement	*p* ^1^	*p* ^2^
Erythema (Median [IQR], mm^2^)	Regular treatment	640.00 (405.79, 916.83)	372.40 (201.37, 635.25)	210.97 (183.78, 322.04)	< 0.001	0.004
	Irregular treatment	727.22 (450.02, 1018.05)	644.28 (362.60, 935.51)	43.09 (37.54, 142.72)	0.012	
Erythema concentration (Median [IQR], %)	Regular treatment	8.08 (6.45, 9.84)	5.97 (4.34, 7.88)	2.09 (1.94, 2.26)	0.003	0.032
	Irregular treatment	9.31 (7.23, 12.92)	8.88 (6.85, 11.93)	0.60 (0.48, 0.82)	0.045	
Hyperpigmentation (Median [IQR], mm^2^)	Regular treatment	577.54 (471.89, 663.60)	370.61 (268.47, 472.88)	192.64 (160.54, 208.68)	< 0.001	0.015
	Irregular treatment	507.99 (101.33, 671.61)	419.69 (68.37, 598.88)	54.40 (35.48, 80.98)	0.023	
Hyperpigmentation concentration (Median [IQR], %)	Regular treatment	7.62 (6.37, 9.16)	5.75 (4.53, 6.95)	2.06 (1.95, 2.16)	0.001	0.028
	Irregular treatment	7.90 (6.46, 9.72)	7.58 (5.90, 9.24)	0.55 (0.51, 0.68)	0.038	
Wrinkle index, Median [IQR]	Regular treatment	216.60 (176.75, 267.25)	95.00 (79.50, 111.25)	123.50 (81.50, 167.00)	< 0.001	0.009
	Irregular treatment	201.00 (134.50, 265.75)	82.50 (51.50, 100.50)	115.50 (85.00, 172.00)	0.008	

*Note:* *p* ^1^, *p*‐value for comparing between the before and after treatment; *p* ^2^, *p*‐value for comparing change between the two groups.

### 3.3 Subgroup Analysis by Diagnosis

**FIGURE 2:**
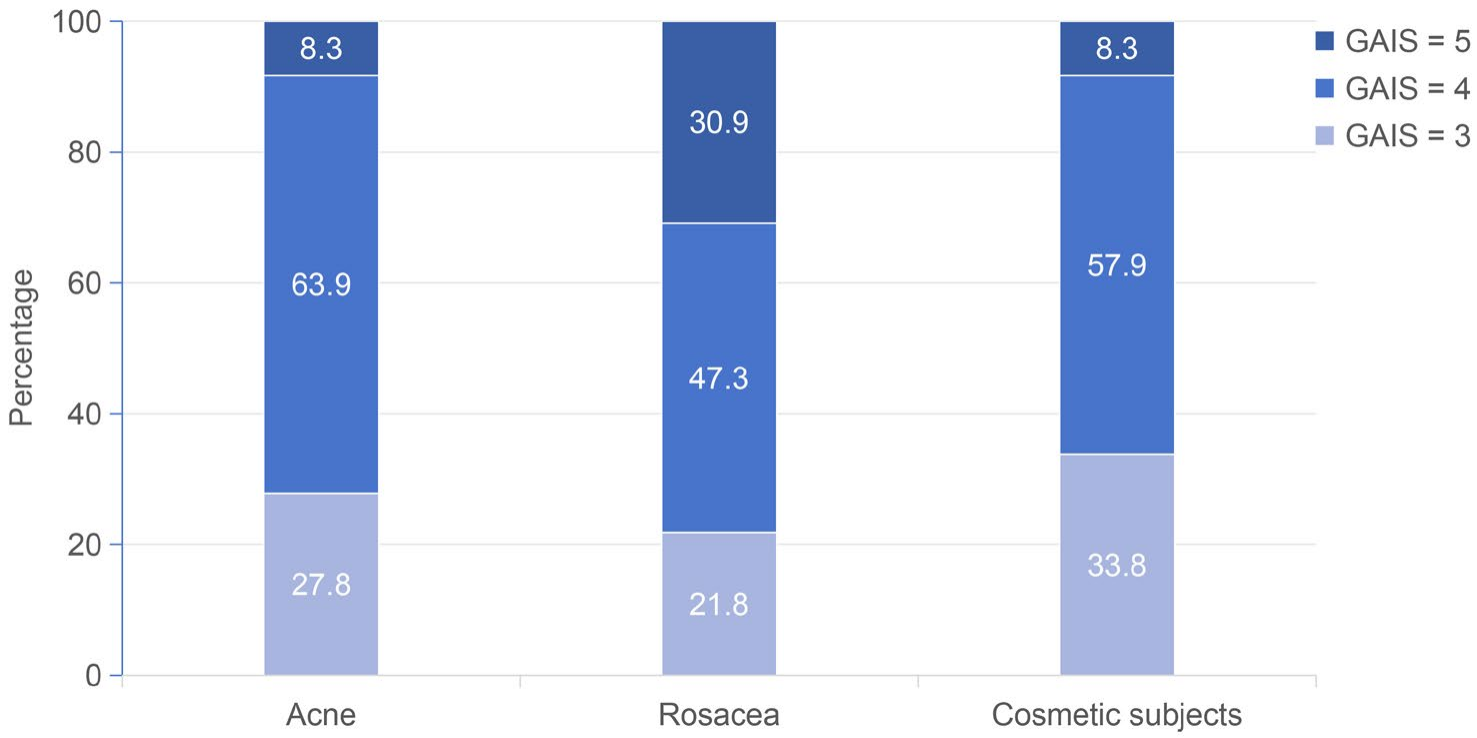
Physicians' GAIS ratings of overall improvement in each patient group post‐treatment (3 = slightly satisfied, 4 = satisfied, and 5 = very satisfied). GAIS, global aesthetic improvement scale.

When analyzed by clinical subgroup (Table 3), rosacea patients exhibited the largest erythema reduction (median change 1820.9 mm^2^ vs. 667.7 mm^2^, *p* = 0.008). Cosmetic subjects demonstrated balanced improvement across all three parameters, while acne‐associated erythema and textural irregularities also showed significant decreases (*p* < 0.05). Physician‐rated GAIS distributions are shown in Figure 2, where most regular treatment participants scored 4 (“satisfied”) or 5 (“very satisfied”). Because GAIS was limited to physician ratings due to missing patient‐reported data, dual satisfaction assessment was not feasible.

**TABLE 3:** Quantitative data analysis of all participants before and after treatment.

	Group	Erythema improvement (Median [IQR], mm^2^)	Erythema concentration improvement (Median [IQR], %)	Hyperpigmentation improvement (Median [IQR], mm^2^)	Hyperpigmentation concentration improvement (Median [IQR], %)	Wrinkle index improvement, median [IQR]	Proportion of good responders with > 6 treatments sessions, *n* (%)	Proportion with regular treatment in good responders, *n* (%)
Acne	Good responders	53.08 (39.13, 234.62)	1.22 (1.17, 1.95)	14.54 (9.3, 22.21)	1.76 (0.84, 2.24)	67.00 (44.00, 84.00)	11 (37.9)	19 (65.5)
	Poor responders	39.32 (31.69, 168.39)	1.07 (0.71, 1.40)	21.61 (8.58, 124.43)	0.66 (0.55, 0.98)	82.00 (62.50, 88.00)	0	2 (28.6)
	*p*	0.032	0.041	0.028	0.035	0.019	—	< 0.001
Rosacea	Good responders	1820.90 (967.87, 2364.96)	2.24 (2.11, 2.41)	205.00 (190.20, 215.91)	2.09 (2.03, 2.20)	159.00 (98.00, 195.00)	14 (31.1)	41 (91.1)
	Poor responders	667.7 (538.35, 863.72)	0.80 (0.73, 2.04)	91.23 (76.59, 109.10)	0.62 (0.51, 0.87)	180.00 (108.75, 197.25)	0	2 (20.0)
	*p*	0.008	0.011	0.006	0.009	0.013	—	< 0.001
Cosmetic subjects	Good responders	204.36 (179.11, 215.98)	2.06 (1.90, 2.13)	192.43 (174.11, 202.68)	2.05 (1.95, 2.12)	127.00 (88.25, 158.5)	21 (21.9)	94 (97.9)
	Poor responders	42.30 (38.96, 46.60)	0.49 (0.44, 0.58)	54.38 (46.35, 73.35)	0.54 (0.51, 0.55)	140.00 (111.00, 172.00)	2 (4.0)	26 (53.1)
	*p*	0.045	0.052	0.038	0.049	0.041	0.003	0.005

*Note:* Participants were cosmetic subjects without a history of facial skin disorders but with subjective concerns regarding skin redness, uneven pigmentation, or texture. GAIS, Physician‐rated aesthetic improvement. Good responders (GAIS = 4–5), poor responders (GAIS 1–3). *p*‐value for comparing improvements between the good responders and poor responders.

### 3.4 Predictors of Treatment Response

Multivariate logistic regression (Table 4) identified three independent predictors of favorable response (GAIS ≥ 4): regular treatment (OR = 13.62, 95% CI: 3.58–51.87, *p* < 0.001), total sessions (OR = 3.80, 95% CI: 1.37–10.53, *p* = 0.010), and Fitzpatrick Type IV skin (negative predictor, OR = 0.12, 95% CI: 0.04–0.42, *p* = 0.001). Age, gender, and diagnosis were not significant predictors. The model achieved an AUC of 0.922, indicating excellent discrimination.

**TABLE 4:** Multivariate logistic regression identifying predictors of good response (GAIS ≥ 4) to IPL treatment.

Variable	OR	95% CI	*p*
Gender	1.599	(0.338, 7.553)	0.554
Skin type, Type IV vs. Type III	0.121	(0.035, 0.424)	0.001
Diagnosis, acne vs. cosmetic subjects	5.439	(0.849, 34.826)	0.074
Diagnosis, rosacea vs. cosmetic subjects	1.671	(0.381, 7.335)	0.496
Regular treatment	13.621	(3.577, 51.865)	< 0.001
Age	1.078	(0.964, 1.205)	0.186
IPL_number	3.796	(1.368, 10.530)	0.010

*Note:* Error bars represent 95% confidence intervals. Model performance: AUC = 0.922; McFadden's *R* ^2^ = 0.532. A vertical dashed line indicates the null value (OR = 1). Variables with *p* < 0.05 are marked with an asterisk.

### 3.5 Safety Outcomes

No serious adverse events were reported. Transient erythema and edema were the most common minor effects, resolving spontaneously within 48 h. No cases of scarring, blistering, or prolonged hyperpigmentation occurred, confirming the safety of IPL when applied under standardized parameters.

## 4 Discussion

This real‐world investigation provides strong evidence that long‐term, regular IPL therapy achieves comprehensive facial rejuvenation by improving erythema, pigmentation, and wrinkles simultaneously. Our data indicate that treatment regularity and cumulative exposure are key determinants of outcome quality, underscoring the importance of structured phototherapy scheduling.

Mechanistically, IPL exerts its effects through selective photothermolysis of hemoglobin and melanin, coupled with mild thermal stimulation of dermal fibroblasts. This triggers vascular remodeling, reduces erythema, and promotes neocollagenesis for textural enhancement [13]. Pigment improvement likely results from melanin fragmentation and accelerated epidermal turnover [14]. These complementary mechanisms explain the synchronized enhancement across the three major dimensions observed in this cohort.

Compared with prior short‐term studies, our data offer a longitudinal perspective that highlights the cumulative benefits of regular IPL sessions. The clear correlation between treatment regularity and efficacy supports the concept of maintenance photorejuvenation. Repeated light exposure at optimal intervals maintains endothelial injury cycles and fibroblast activation, yielding stable collagen deposition and long‐term improvement [15, 16].

The less pronounced efficacy in Fitzpatrick Type IV skin is consistent with established optical absorption models. Melanin's higher absorption coefficient in darker skin necessitates conservative fluence settings to prevent adverse effects, inadvertently reducing energy delivery to vascular and dermal targets [17, 18]. For such patients, extended intervals and wavelength optimization may balance efficacy and safety [17].

Importantly, our study demonstrates IPL's reproducibility under routine practice conditions, supported by quantitative VISIA imaging. Objective imaging reduces subjectivity, allowing standardized measurement of erythema, pigmentation, and wrinkles [8]. These findings reinforce IPL's role as a multidimensional, evidence‐based modality for holistic facial rejuvenation.

However, the absence of patient‐rated GAIS data limits interpretation of subjective satisfaction. As this was a retrospective study, inconsistent follow‐up documentation precluded comprehensive patient feedback collection. Nevertheless, physician‐rated GAIS remains a validated metric and correlates well with objective imaging outcomes (Table 3, *p* < 0.05).

### 4.1 Clinical Implications

The findings provide a practical framework for clinicians to optimize IPL regimens. Regular sessions maximize cumulative effects and minimize relapse of vascular or pigmentary lesions. VISIA imaging can be incorporated into practice to objectively monitor response, personalize fluence parameters, and reinforce adherence. This approach enhances treatment predictability, patient trust, and long‐term satisfaction.

### 4.2 Limitations

Several limitations merit consideration. The retrospective design introduces potential selection bias and limits causal inference. Since doctors will make personalized adjustments based on the patient's condition before the IPL begins, treatment parameters such as fluence and filter selection were not fully standardized, which may influence reproducibility. Additionally, only physician‐rated GAIS scores were analyzed, as patient‐reported data were incomplete; however, physician evaluations are considered objective and reliable in retrospective contexts. Future prospective studies incorporating standardized treatment protocols and dual GAIS assessments are warranted to validate and expand these findings.

## 5 Conclusion

Long‐term, regular IPL therapy achieves significant, multidimensional facial rejuvenation encompassing erythema reduction, pigment correction, and wrinkle improvement. Adherence to structured treatment intervals and sufficient session completion are essential for optimal outcomes. The integration of VISIA‐based imaging with clinical evaluation establishes IPL as a robust, evidence‐based approach for sustained skin rejuvenation.

## Author Contributions

Dr. Fan collected and analyzed data, and drafted the manuscript. Prof. Yu provided key data and critically revised the manuscript.

## Funding

Supported by the National Natural Science Foundation of China (Grant No. 82574002) and Elite Medical Professionals Project of China‐Japan Friendship Hospital (NO. ZRJY2024‐QMPY03).

## Ethics Statement

Ethical approval was obtained from the Institutional Ethics Committee of China‐Japan Friendship Hospital (Approval No. 20251011LC). All procedures followed the ethical standards of the Declaration of Helsinki.

## Consent

The written informed consent for photographs and/or video images was signed by all subjects.

## Conflicts of Interest

The authors declare no conflicts of interest.

## Data Availability

The data that support the findings of this study are available on request from the corresponding author. The data are not publicly available due to privacy or ethical restrictions.
